# Equivalent Pathways? Comparison of Job History for High School Graduates Versus GED Recipients

**DOI:** 10.1093/geroni/igaa057.1959

**Published:** 2020-12-16

**Authors:** Wenxuan Huang

**Affiliations:** CASE WESTERN RESERVE UNIVERSITY, Cleveland, Ohio, United States

## Abstract

Successful integration into the paid labor market serves as a critical milestone to adulthood. Yet, this school-to-work transition has become harder to reach due to the increasing precarity in the youth labor market. Using data from the NLSY97, this study compares the job histories of young adults whose terminal education credentials are high school diploma versus GED. I conducted sequence analysis of school-to-work states from age 16 to 30 between these two education groups. Findings show that GED holders are more likely to be exposed to enduring negative labor force status (e.g., periods of unemployment) than the high school graduates. Over half of the GED recipients experience precarious early career characterized by interruptions and long-term inactivity. Despite being “equivalent” to a high school diploma, the GED diploma does not translate into the same opportunity structure as the high school degree, launching a cumulative disadvantage process in the early life course.

